# Beyond plans, governance structures, and organizational strategies: how emotional mechanisms can make a difference in emergency response processes

**DOI:** 10.1007/s11077-022-09480-4

**Published:** 2022-11-13

**Authors:** Stefania Ravazzi

**Affiliations:** University of Torino, Torino, Italy

**Keywords:** Emergency policy, Emotions, COVID-19, Robustness

## Abstract

Emergency policies are among the most challenging policies that policy makers have to deal with, because of their extreme seriousness, the lack of time, and the high uncertainties that are involved. Policy analyses have demonstrated that good structural and organizational strategies are important, but not sufficient, to systematically guarantee a high level of resiliency in response processes. Some scholars have therefore suggested the need to verify whether individual cognitive and relational mechanisms can contribute to explaining the different levels of resiliency that emerge in emergency response processes. From such a perspective, this article presents the findings of a research that was aimed at testing whether emotional mechanisms matter. The affect infusion model was used to provide the analytical framework that was considered to identify the evidence necessary for the empirical research, and the ‘most similar system design’ was applied to select and compare two couples of emergency response processes with similar contextual, structural and organizational features, but different levels of resiliency. The empirical research was conducted from April 2020 to February 2021, through periods of job shadowing and semi-structured interviews with personnel from the public and private organizations involved in the response processes. The research has substantially corroborated the hypothesis and has highlighted that, despite very similar contextual, structural and organizational conditions, a negative emotional mechanism, triggered by fear and anxiety, was pervasive among managers involved in the two lower-resiliency emergency response processes, while a positive emotional mechanism, triggered by pride, was dominant among managers involved in the two lower-resiliency processes.

## Introduction

The interest of policy analysts in emergency situations and the consequent response policies has increased over the last few decades. The reason for this is not only because natural disasters, terrorist attacks, energy blackouts, nuclear accidents, oil spills, financial crises and epidemics have become more frequent, but also because turbulent events have become more and more transboundary, and their effects are more and more difficult to predict (Rosenthal, Charles and t’ Hart, 1989; Ansell et al., 2010, 2021).

One of the most topical issues in the debate on emergency policies concerns what factors and strategies can favor resilient and robust policy making (Kendra and Wachtendorf, 2003; Ansell et al., 2021). According to Capano and Woo (2017), resilience has been considered in policy analysis to mean the ability of social systems to bounce back to their previous equilibriums after a shock or perturbation. Robustness instead means the ability of organizations or systems to withstand unexpected changes and uncertainty by changing their structures and processes while preserving their primary functional characteristics. Robustness is today considered a key concept in policy analysis, which is mainly composed of three dimensions: *creativity*, *agility* and *collaboration* (Ansell et al., 2021). But what actually favors robustness among policy actors and among organizations in emergency situations?

Although most studies on emergency policies have not addressed the issue of robustness directly, they have highlighted some factors as being of fundamental importance to improve response policies to emergency situations. The first factor concerns the multi-level governance system, which is often described by distinguishing between bottom-up and top-down systems. In the former case, local governments are in the front line of emergency management actions, while upper-level governments intervene only subsidiarily, by coordinating lower-level governments and by supporting them with resources and personnel when necessary. In the latter case, central level governing bodies intervene to directly manage the emergency, thus taking the place of lower-level governments in several decisions and actions. According to several scholars, a bottom-up governance system improves response policies to emergency situations (Schneider, 2011; Waugh, 2000), but it is difficult to put in place, because a certain degree of confusion is usually present, and bottom-up and top-down interventions end up coexisting together in messy processes, especially in the case of particularly serious turbulent events (Birkland, 2009b; Comfort and Kapuku, 2006; Kapuku, 2009; Ansell et al., 2010; McGuire & Agranoff, 2011). The second factor is the availability and application of clear and detailed operative action guidelines (evacuation or quarantine protocols, warning and communication procedures, mutual aid and funding rules, etc.) (Miccoli & Destefano, 2010; Van Eeten et al., 2011; Fischbacher-Smith and Fischbacher-Smith, 2016), even though they are usually only partially applicable and can sometimes even end up prefiguring ‘improbable missions’ (Clarke, 1999; Boin, Comfort, and Demchack, 2010). The third factor concerns the characteristics of the problem-solving teams that are usually created to steer and coordinate emergency operations at the upper level. Emergency management actions seem to improve when central problem-solving teams are multi-expertise, cohesive, and relatively small (Arendt & Alesh, 2015), although empirical studies have demonstrated that such teams can lack certain essential expertise for political or contingent reasons and can miss some information, which is never perfectly controllable (Schneider, 2011).

Recently, some studies, although not denying the importance of preparing accurate emergency management plans and moving toward bottom-up management systems coordinated by small, central, multi-disciplinary teams, have turned their attention to relational aspects as a crucial explanatory dimension (Ansell et al., 2010; Legrand and McConnell, 2011; Arendt & Alesh, 2015; Boin & van Eeten, 2016; Kapucu and Boin, 2016). From this perspective, emotional mechanisms are in particular expected to play a role, since human decisions and behaviors in the face of particularly challenging situations are influenced to a great extent, not only by reflexive thinking, but also by emotion schemas, namely dynamic interactions between emotions, perceptions and cognitive and relational processes (Izard, 2009; Kahnemann, 2011; Legrand & McConnell, 2012; Lerner et al., 2015). The aim of this article is to contribute toward explaining the role of emotional mechanisms in emergency response policies. In particular, the article tests the hypothesis that counter-productive emotional mechanisms, triggered by fear and anxiety, can contribute to lowering the robustness of response policies, while positive and productive emotional mechanisms, triggered by pride, can contribute to increasing the robustness of such response policies.

In order to test this hypothesis, I conducted empirical research on the main response policies to the COVID-19 emergency in Italy. The COVID-19 pandemic has affected most countries throughout the world, forcing them to face emergency challenges they had never experienced before, but Italy was affected more by the contagion than any other European country in the first wave of infection and, in just a few weeks, was almost completely isolated from the rest of the world, with a huge shortage of ventilators, medicinal supplies, personal protection equipment, as well as personnel and healthcare spaces, in the middle of a silent war on the procurement of life-saving goods.^1^ I carried out the empirical research from April 2020 to February 2021, through the conduction of six job shadowing periods with the staff of the Special Commissioner for Emergency Management, who had been appointed by the Prime Minister to lead and coordinate the most important operations,^2^ the direct observation of 10 operative meetings, the analysis of 96 min of Technical and Scientific Committee meetings and 43 official documents on response operations, and through the conduction of 118 semi-structured interviews with the personnel of the public and private organizations (national and regional departments, hospitals, public agencies, firms, professional associations, NGOs) involved in the major policy-making processes put in place to respond to the first and second waves of the infection. The interviews were focused on episodes and events that the individuals had experienced, their decisions and actions, their feelings, and their considerations about what was unsatisfactory or what was somehow working well and could represent an element of learning. Among all the response policies that I observed and analyzed during the research, I selected two of them to allow a comparison to be made of processes with similar characteristics and conditions but different degrees of robustness (41 interviews out of 119 concerned these two policies^3^). The decision to analyze the narratives, interpretations, and perceptions of individuals was in line with other scientific research activities that used self-reported semantic terms, narratives, and explanations of subjective experiences to detect emotions and emotion schemas. This qualitative approach is considered a more neutral strategy than using predefined survey items, and the only feasible method to analyze individual attitudes toward real ongoing real circumstances instead of imaginary situations in laboratory experiments (Barrett et al., 2007; Cowen & Keltner, 2017; Ledoux et al., 2016; Robinson & Clore, 2002; Tsuchiya & Adolphs, 2007).

The paper is structured as follows. The next section frames the issue of robust policy making and the role of structural and organizational factors in emergency response policies. Section 2 introduces the role of emotion schemas in decisional and operative processes and explains the two main hypotheses considered in the empirical research. The third section describes the main features of the response policies to the COVID-19 emergency in Italy during the first and second contagion waves, and in particular the ones that were considered for the analysis. The subsequent two sections explain the main findings of the research, while the Conclusion section synthesizes the main arguments presented in the article.

### Emergency response policies and the robustness issue

The effectiveness of human responses to turbulent events and turbulent periods is still weak, and policy analysts are still trying to understand the reasons for this weakness (Schneider, 2011). Nevertheless, the literature on emergency policies has progressively created an explanatory framework to account for the higher or lower robustness of emergency policies.

An emergency policy is composed of four main intervention categories: mitigation, preparedness, response, and recovery. In practice, all these policies overlap to a certain extent, but they also differ in some respects. Mitigation and preparedness policies are in general antecedent to a turbulence. The focus of mitigation policies is ‘to prevent hazards from developing into complete disasters or to reduce the effects of disasters when they occur’ (Chu, 2011, p. 451). Some examples of such policies are: creating channels for volcanic debris flows, building dams, riverbanks, or wildfire corridors. Preparedness policies are composed of measures that are aimed at preparing people and organizations to react to hazardous events promptly, by forecasting the probability of future risky events and by recognizing alarming situations before the turbulence occurs (Handmer & Dovers, 2007; Fischbacher-Smith and Fischbacher-Smith 2016). Some examples of preparedness policies are training programs for emergency managers and drills. Emergency response policies take place while the shock or crisis is happening and immediately after, and they are aimed at reducing damage, avoiding disasters or catastrophes and at securing (at least partially) the provision of services and public goods (Schneider, 2011). Early evacuations, rescue operations and the special purchase and distribution of goods are all examples of these policies. Recovery policies usually take place after the severest phase of the emergency has been overcome (Kendra and Wachtendorf, 2003; Haddow et al., 2007), and are put in place in order to recover from the consequences and ripple effects of a turbulence and to build a new normality, possibly avoiding reproducing past vulnerabilities and trying to take a step forward toward the mitigation of future disasters (Arendt & Alesh, 2015). The reconstruction of streets and buildings after earthquakes or hurricanes and support for the renewal of social and economic activities are some examples of this latter type of emergency policy.

Response policies are the most challenging and stressful type of emergency policies (Handmer & Dovers, 2007; Ross, 2014). The lack of time, which may also characterize many policies in normal periods, becomes dramatic under such conditions (Arendt & Alesh, 2015, p. 189; Kapucu and Boin, 2016), and any changes in the problem and/or the situations, which are always present in policies, become sudden and pervasive (Clarke, 1999; Handmer & Dovers, 2007). Finally, responses to disasters and crises fall under the spotlight far more than mitigation, preparedness, and recovery policies (Schneider, 2011), thereby increasing the pressure and stress for the people involved in the emergency management activities. Under these difficult conditions, special efforts are necessary to attain *robustness* in policy making (Erbeyoglu & Bilge, 2020).

According to Ansell et al. (2021), the concept of robustness was first used in the biology and engineering fields to define the ability of a system to put in place prompt actions that are able to deal with a shock in ways that uphold the main functions of the system. This concept has also been borrowed by social scientists in such fields as policy analysis and design (Anderies & Janssen, 2013; Capano & Woo, 2017, 2018; Howlett et al., 2018) and economics (Leeson & Subrick, 2006), as a property of individuals, organizations, processes, and social systems. On the whole, robustness in policy processes seems to mainly revolve around three key properties: *creativity*, *agility* and *collaboration.* Creativity can be defined as the capacity to find original solutions and to put in place innovative and flexible operative actions instead of mechanically applying protocols and codified procedures. Agility can be defined as the capacity to change solutions and adapt operative processes to an occurrence, in the case of exogenous or endogenous events. Collaboration can be defined as the alignment of different action logics and operative perspectives to common goals in order to coordinate people and organizations effectively (Ansell, Sørensen and Torfing 2010; Christensen & Laegreid, 2020).

These three properties are as obvious in theory as they are difficult to produce in reality (Boin & van Eeten, 2016): creativity and agility can be undermined by all the well-known drifts and biases that characterize bureaucrats in public and private organizations, such as routinization, resistance to change, logic of appropriateness and goal heterogenesis (Handmer & Dovers, 2007); coordination and collaboration are difficult to achieve when the people involved in emergency management operations are in conflict about the interpretation of goals, rules, procedures, and roles, or when emergency units jostle with each other for resource supplies and/or funds (Tierney, Lindell and Perry, 2001; Schneider, 2011). It is therefore of fundamental importance to understand what can favor these properties in emergency response processes.

The availability of clear, detailed, and updated emergency response plans can significantly help in the collaboration and coordination between individuals and organizations, if the plans assign simple and clear roles and responsibilities to governments, organizations, and people, and establish what everyone should do, when and how (Waugh, 2000). In order to reach this ideal goal, different plausible disaster scenarios are usually formulated carefully by emergency response planners, and the most effective response model is then designed through various simulations (Erbeyoglu & Bilge, 2020; Moore & Lakha, 2011). Some emergency plans even have the aim of considering the ‘potential crisis portfolio’ (Fishbacher-Smith and Fishbacher-Smith 2016, p. 33) of the organizations that could be affected by the emergency operations, namely the gaps between their actual procedures and the operations that could be necessary during such a disaster, their difficulties in managing information flows and information processing about risks, their interactive complexity due to cascade failures, and their internal personnel risks. On the basis of this information, and in light of the simulations conducted about future risky events, emergency response plans (flood management plans, epidemic management plans, earthquake management plans, etc.) offer action guidelines to a large number of policy makers and managers in public and private organizations (Clarke, 1999; Handmer & Dovers, 2007; Schneider, 2011; Waugh, 2000).

The setting up of a bottom-up governance system, that gives the responsibility of planning, organizing, and implementing the rescue operations and all the other response policies to local and regional governments in collaboration with other public and private organizations, in particular fosters the agility of organizations and processes (Schneider, 2011). Such a system also requires intervention at a national or federal level, but with guidelines, funds and personnel for aid and assistance *through*, not *in place of*, lower-level governments and following the voluntary request of lower-level governments. National or federal governments in many countries have often intervened and tried to put in place such a governance system (Kapuku & Boin, 2016; Schneider, 2011).

The coordination of the bottom-up governance system by small, central, ad hoc problem-solving teams, composed of people with different kinds of expertise, knowledge and competence, fosters creativity, and collaboration, if a multi-expertise composition of the teams is effectively realized and if extraordinary powers and room for maneuver are granted to them. These features allow these teams to count on direct information about different policy processes, to integrate different kinds of knowledge, and to intervene promptly in the case of conflicts arising among lower-level governments and between public and private actors involved in response operations (Arendt & Alesh, 2015; Waugh, 2000). Over the last few decades, some emergency response policies have effectively been steered and coordinated by teams with these characteristics at the national, regional, and local levels (Arendt & Alesh, 2015).

Although these structural and organizational features are essential and complementary to each other, they are never present in pure or optimal forms, and organizations and processes often suffer from confusion, ambiguities, stalemates, and from slowdowns. Plans are usually hyper-rational and apply highly improbable assumptions to allow for the formulation of detailed response guidelines (Birkland, 2009b). The consequence is that emergency response actions are always full of deviations from what is established in such documents (McConnell, 2011). Moreover, training programs for emergency managers, which are in general based on these plans and share this hyper-rational approach, only seem to be useful for relatively simple and standard response policies, such as small evacuation procedures, and are insufficient to develop effective action models for complex emergency situations (Handmer & Dovers, 2007; Schneider, 2011). This is why some experts have labeled emergency plans ‘fantasy documents,’ namely ‘symbols to signal that organizations are in control of danger, whether they really are or not’ (Clarke, 1999, 16). Well-structured bottom-up governance systems often become partially confused and generate conflicts of attributions and stalemates (Schneider, 2011), because of bad competition mechanisms between local governments about receiving funds and personnel from the national or federal government, and because of systematic lag-effects of the upper-level government interventions. Moreover, lower-level governments and organizations may be so weak in the most severe disasters that they are unable to handle the emergency, and they often need the direct intervention of the upper-level governments. As a consequence, even within highly decentralized governance systems, national and federal governments may end up intervening directly in emergency operations with various top-down actions (Birkland, 2009a; Comfort and Kapuku, 2009; Kapuku, 2009; Ansell et al., 2010; McGuire & Agranoff, 2011; Christensen & Laegreid, 2020). Small, central, multi-expertise and cohesive problem-solving teams sometimes end up misinterpreting information or lacking internal cohesion, so that violations of standards, procedural failures, delays, and deficits occur frequently, even in the so-called High-Reliability Organizations (nuclear plants, air traffic control centers or space agencies), in which such teams are structurally and organizationally well prepared to fulfill high-risk functions on a day to day basis (Boin & van Eeten, 2016).

**Fig. 1:**
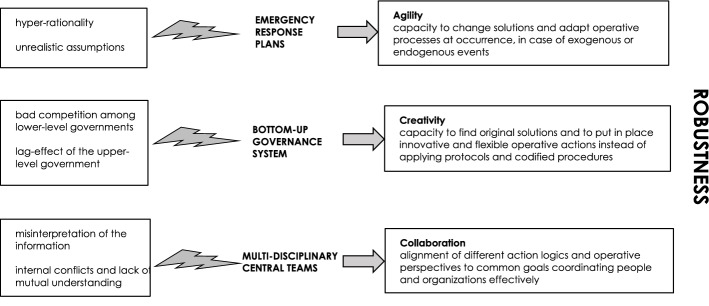
Structural and organizational factors for robust emergency response policies

Figure 1 summarizes the three robustness dimensions and the organizational and structural factors that are important in emergency policies.

In light of the aforementioned considerations, some scholars have suggested integrating the structural and organizational perspectives with cognitive and relational perspectives, in the belief that cognitive processes and interpersonal relations can also contribute to explaining lower or higher levels of creativity, agility, and collaboration in response policies (Arendt & Alesh, 2015). This article adopts this perspective, and focuses in particular on the emotional dimension, in order to understand whether specific emotional mechanisms can contribute toward explaining higher or lower robustness in response policies.

### Emotional mechanisms and emergency response processes

Policy analysists are used to analyzing cognitive mechanisms in policy making, but emotional dynamics has been neglected for decades, in spite of the fact that Herbert Simon (1983) recognized that emotions play a fundamental role in human decisions. Cognitive and social psychologists have long demonstrated that ‘emotions powerfully, predictably, and pervasively influence decision making’ (Lerner et al., 2015, p. 802). It has been highlighted, in psychological literature, that emotional mechanisms are more powerful when people believe they have insufficient knowledge about the problems at stake, when predicting even upcoming events is almost impossible, and when decisions must be made very rapidly, all aspects that are typical of emergency situations (Frijda, 1988; Legrand & McConnell, 2012; Levenson, 1994; Oatley & Jenkins, 1992). Emotions have begun to attract the attention of policy analysts in recent years, but the role of emotions in policy making has so far mainly been framed as a negative interference with rationality, since most studies have focused on misjudgments, mistakes, and nonproportional policy responses to public problems generated by non-rational thinking (Koehler and Harvey, 2004; Thaler & Sunstein, 2008; Vigoda-Gadot & Meisler, 2010; Maor, 2012, 2014).

This article proposes a different perspective, that is, it assumes that ‘whether a specific emotion ultimately improves or degrades a specific judgment or decision depends on interactions between the cognitive and motivational mechanisms triggered by each emotion’ (Lerner et al., 2015, p. 816) and that emotions can positively affect policy making, even in extremely challenging situations, such as disasters and crises.

The affect infusion model (AIM) treats emotions in a context of specific cognitive processes and interpersonal predispositions (Izard, 2009; Keltner & Lerner, 2010). According to this model, individuals usually formulate very simple and fast initial appraisals of the external situation when they have to make decisions and act. These appraisals generally suggest how much attention and energy should be invested to deal with the situation with respect to personal aspirations, or, in other words, the level of personal commitment that it deserves (Smith & Ellsworth, 1985). This rapid and intuitive evaluation triggers emotional reactions, such as affects, moods, and feelings, which manifest themselves through neural and physical symptoms and can more or less be identified and framed by individuals (Janig, 2003). In turn, emotions trigger different cognitive processes as they affect certain specific cognitive dimensions: control, namely the degree to which the individual believes events are brought about by his/her agency versus situational agency; certainty, namely the degree to which the individual believes future events are predictable and comprehensible, or not; attribution of responsibility, namely the degree to which someone or something other than oneself versus oneself is considered responsible for the situation; and legitimacy, namely the extent to which the situation is fair or unfair (Lerner & Keltner, 2000, 2001; Rottenstreich & Hsee, 2001; Loewenstein et al., 2003; Denhardt & Denhardt, 2006; Newman et al., 2009; Lerner et al., 2013, 2015; DeSteno et al., 2014). Emotions also affect interpersonal predispositions, in particular the aptitude for listening to or for resisting information and the opinions or needs of others, the aptitude toward compromise or intransigence, namely the predisposition to give or deny concessions, and the aptitude toward hostility or mildness, namely the predisposition to behave aggressively or in a friendly way (DeSteno, 2009; Rind & Bordia, 1995; Van Beest et al., 2008; Van Kleef et al., 2010). Cognitive processes and interpersonal predispositions eventually affect the formulation of goals, and the choice of means and concrete actions (Lerner et al., 2015; Raghunathan & Pham, 1999). Since emotional mechanisms work as dynamic processes of intertwined components instead of as linear chains of stimuli and reactions, they can be self-reinforcing if something (or someone) does not change the dynamics (Izard, 2009).

Cognition and interpersonal predispositions are also affected by personality traits, by ‘incidental’ emotions that arise in the individuals as reactions to other situations^4^ (Bodenhausen, 1993; Loewenstein & Lerner, 2003; Watson & Clark, 1992), and by culture (Ellsworth & Scherer, 2003; Keltner & Lerner, 2010), but in the end the emotions that are triggered in specific circumstances remain powerful, especially in the presence of particularly shocking and impressive situations (Keltner & Lerner, 2010; Legrand & McConnell, 2012).

Different emotions tend to affect cognitive processes and interpersonal predispositions in partially different or even completely opposite ways. Although the boundaries between categories of emotions tend to be fuzzy rather than discrete, some emotions, such as fear, anxiety, anger, discontent, contempt, guilt, or shame, have a clear negative valence, while others, such as quietness, contentment, joy, gratitude, pride, satisfaction or relief, have a clear positive connotation (Cowen & Keltner, 2017; Izard, 2009; Keltner & Lerner, 2010). As far as emergency situations are concerned, the pervasiveness of fear and anxiety can in particular be expected, because of the high uncertainty, the lack of time, and the complexity of the situation, but emergency managers can also be expected to feel something that is similar to pride, because of the intrinsic sense of responsibility of doing something fundamental for the community, and because of the adrenaline rush that arises when working together with very little time and challenging goals.

**Fig. 2:**
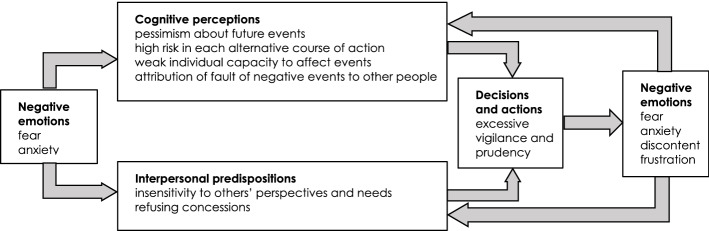
The AIM negative emotion schema triggered by fear and anxiety

According to AIM, fear and anxiety in particular influence the feelings of certainty, control, and attribution of responsibility, as they induce people to formulate pessimistic evaluations of future events, to perceive a high risk of future choices and actions and a weak individual control capacity over the situation, and to attribute the fault of specific negative events to other people (Lerner et al., 2015; Loewenstein & Lerner, 2003). Empirical evidence about the impact of fear and anxiety on interpersonal predispositions is more questionable, but it seems that these negative emotions tend to decrease the aptitude of individuals to make concessions and to increase closure against the requests of others (Lerner et al., 2015). The combination of these cognitive processes and interpersonal predispositions in turn induces individuals to act in overly vigilant and cautious ways, thus triggering a vicious circle of discontent and frustration (Fig. 2).

**Fig. 3:**
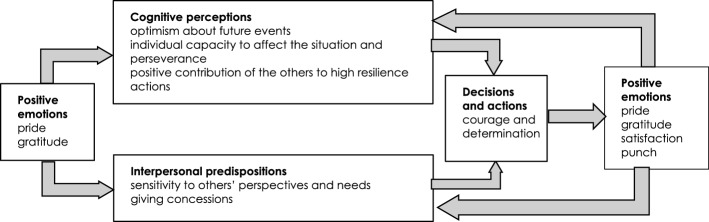
The AIM positive emotion schema triggered by pride and gratitude

Pride seems to affect the cognitive dimensions of control and legitimacy, thus increasing the tendency to perceive positive events as predictable and the direct consequences of personal decisions and actions, and to consider the contribution of others positively (Fredrickson, 1998, 2001; Isen, 1987). Moreover, these positive emotions also seem to trigger positive interpersonal predispositions that are in contrast with the ones triggered by fear and anxiety: people tend to be more predisposed to granting concessions and more inclined to listen to the needs and viewpoints of others. The combination of these cognitive processes and interpersonal predispositions in turn induces individuals to act promptly and courageously, thereby triggering a virtuous circle of satisfaction and contentment (Fredrickson, 1998, 2001; Isen, 1987) (Fig. 3).

In light of this framework, the main thesis of this article is not only that emergency situations can trigger both negative and positive emotional mechanisms, but that their valence is able to affect the way in which emergency managers and personnel involved in response operations act. In other words, the hypothesis arising from the application of AIM is that people who feel extreme fear and anxiety put in place pessimistic cognitive processes and counter-productive interpersonal predispositions that make them decide and act in less creative, agile, and collaborative ways than people who feel intense pride and, consequently, put in place optimistic cognitive processes and productive interpersonal predispositions.

I conducted the analysis by searching for specific evidence in two of the response processes considered in the research. These two processes were characterized by similar structural and organizational factors and by similar challenges, but performed differently in terms of robustness. On the one hand, I hypothesized that the people involved in lower-robustness processes:

Ia felt fear and anxiety as pervasive emotions.
Ib had pessimistic perceptions about the evolution of the situation, considered alternative solutions as equally risky or unfeasible, were convinced they had limited capacities to control the situation and tended to attribute the fault of specific negative events to others;
Ic showed insensitivity to the viewpoints and needs of others and tended to systematically deny concessions to their requests;
Id experienced frustration and discontent as a consequent emotional reaction to interactions with other people involved in the processes.

On the other hand, I expected that the people involved in Italian higher-resilience processes:

IIa felt pride as a pervasive emotion
IIb had optimistic perceptions about the evolution of the situation, were convinced they had good capacities to affect the events, and considered the contribution of others to the response actions positively;
IIc showed sensitivity to the viewpoints and needs of others and a positive predisposition to grant concessions in reaction to the requests of other actors;
IId experienced satisfaction as a consequent emotional reaction to interactions with other people involved in the processes.

### Italy and the COVID-19 emergency

It is impossible to deny that the recent Covid-19 pandemic has created a tremendous turbulence and has required unprecedented emergency measures, at least in the countries that have been affected the most.

From the case-study research perspective (Hague et al., 1998), the Italian situation can be considered as a crucial case for emergency response policies, since it was the first European country to be affected by the epidemic and, consequently, the first that had to design and implement response policies. One month after China reported a cluster of cases of pneumonia of unknown etiology (later identified as a new coronavirus—Sars-CoV-2) in the city of Wuhan, the World Health Organization (WHO) declared that the Chinese coronavirus epidemic had become an international public health emergency. On January 31, 2020, the Italian Prime Minister declared a State of Emergency and the national and regional governments started to adopt the first policy measures. Since then, hundreds of Prime Minister’s decrees, government executive decrees, national and regional laws, and national, regional, and provincial executive orders have been enacted to deal with the spread of the infection. The aim has been to reduce the contagion through extreme restrictions of movements, contacts, and social activities, the facilitation of smart working, the temporary interruption of productive and commercial activities, the organization of a system of distance teaching and learning, the supply of goods and services to protect and cure citizens, and measures to support the economy. After the declaration of a state of emergency, the national government activated the Civil Protection Department (CPD) for some immediate operations, and also instituted a Technical and Scientific Committee (TSC) to advise the government on public health policy measures. The first measures involved blocking flights from China, measuring the temperature of passengers in the main airports and placing sick passengers in quarantine. The CPD worked, in collaboration with civil servants at the national and regional level, with the Army, the Red Cross, hospital managers, airport and port authorities, cruise liner personnel, and hotel managers to organize and manage the quarantines of the people who disembarked onto Italian soil and to erect tents at the entrance of emergency wards and penal institutes for the triage of personnel, patients and convicts. These initial response actions were relatively manageable, since the virus had not yet spread to any significant extent, and most operations had already been experienced during other emergencies in the past. Some weeks later, when the WHO declared that COVID-19 had become a pandemic and the diffusion of the contagion in Italy had worsened drastically, the national government gave the order to start repatriation procedures for Italian citizens who were living, working or studying temporarily in other countries, and to close schools and universities.

But Italy can also be considered as a representative case for emergency response policies. Firstly, although none of the involved organizations proved to be really prepared for such a devastating and unknown contagion—in the same way as in all the other countries, except South Korea (Lee et al., 2020)—emergency plans containing guidelines on how to face flu epidemics were already available for national and regional managers.^5^ At the same time, a bottom-up governance system was set up, in which regions directly managed the emergency operations, while the national government intervened by introducing legal frameworks and programs, providing additional funds, life-saving goods and personnel, and coordinating regional governments in order to limit fund rush and spillover effects. However, the national government had to intervene directly and massively on several occasions to support overwhelmed and exhausted regional governments (Casula et al., 2020). In just a few days, the need for personal protection equipment, ventilators, and for specific medicinal supplies for patients increased dramatically, the intensive care units of many hospitals rapidly became full, and the ambulance services and emergency wards were soon overwhelmed and unable to deal with all the emergency calls. Thus, improvised transfers were organized to move patients from health structures that could not host them, and more and more protective face masks, visors, gloves, gowns, and overshoes were ordered to protect the healthcare personnel. However, the magnitude of the contagion and the graveness of the disease soon became evident, and the number of dead began to increase exponentially. The highly regionalized national health system risked collapsing and disaster was around the corner. At that moment, that is on 9 March, the Prime Minister declared the first full lockdown in the country and, on 17 March, appointed a Special Commissioner for Emergency Management (SCEM) with the task of coordinating the response policies and supporting the public and private actors involved in the management of the emergency. The Commissioner was provided with special funds and with a dedicated staff composed of advisors, managers, and employees with different expertise. The features of SCEM were similar to those of the high-level problem-solving teams that are usually instituted to lead emergency management operations, that is, an ad hoc structure with a flexible and heterogeneous composition and diversified competences, at the center of all information flows and in constant connection with all the public departments and agencies involved in the emergency management task. The most complex response processes during the first and second waves of the contagion were coordinated and/or supported by SCEM: the procurement and distribution of ventilators, the procurement of personal protection equipment, the importing of medicinal supplies and oxygen, the repatriation of Italian students and workers from other countries, the construction of new, equipped spaces in hospitals for intensive and semi-intensive care, the procurement of single-seat desks for the reopening of primary and secondary schools with the required security distance, the recruitment of new doctors and nurses and of new teachers and auxiliary personnel, the development of the contact-tracing technology and procedures, and the procurement of swabs and reagents to create a surveillance system on the spread of the infection. These processes involved complex networks of public and private policy makers, at a national, regional, and local level, with different, legal/administrative, technical/scientific, and managerial/strategical expertise.

Some of these emergency response processes proved to be more robust than others. The two main procurement policies shared similar objectives and operative challenges, but differed substantially in their robustness: the procurement of personal protection equipment for healthcare personnel and the population in general, and the procurement of single-seat desks for the reopening of the primary and secondary schools. Both procurement policies took place over several months and their emergency management networks, in addition to involving central coordination by the SCEM staff, technical supervision by the TSC and the operative support of the CPD staff at the national and regional level, also included civil servants from different ministerial and regional departments, firms, local public service institutions with high organizational autonomy (hospitals and laboratories in the former case, schools in the latter), trade unions, professional associations and trade associations. Moreover, the complexity of the issues at stake was similar, and the challenges for the production systems were comparable (unprepared markets, technical difficulties for the conversion of companies to new production lines and distribution constraints). Finally, the salience of the issues was high in both cases: although the procurement of single-seat desks did not have the connotation of being a life-saving policy, while the procurement of personal protection equipment did, the need to reopen schools in safe conditions had become a hot topic and was perceived as an absolute priority, because of the perceived serious learning and socialization problems of children and teenagers. However, the personal protection equipment procurement policy proved to be more robust than the single-seat desk procurement policy: most of the former policy actors showed more creativity in facing the numerous operative challenges and obstacles, the implementation processes proved to be more agile in face of the continuously changing context, and more collaboration and coordination emerged between the different government levels and public and private actors.

### The procurement of single-seat desks: low robustness and negative emotional mechanisms

The education system had to be adapted to create distance learning through the improvement and development of school information and communication infrastructures, on which efforts were focused for several weeks. However, the main challenge for the education system was represented by the rules put in place for their reopening, which required not only extraordinary sanitization procedures to be planned and personal protection equipment to be procured for students and school personnel, but also the spaces to be reorganized to respect the two-meter interpersonal distance required by the TSC. Some schools did not have enough space in the classrooms to guarantee the required distance and had to find new spaces where they could move students, while some schools were only equipped with two-seat desks and had to renovate their desk stocks to allow the students to attend. The time available to address these problems was limited, and the government tried to face these challenges while helping schools to shift to distance teaching and learning. The CPD was asked to help schools and local governments find new spaces by adapting halls and gyms or by finding other buildings that could be used temporarily. The Direction for human and financial resources of the Ministry of Education had to recruit more personnel and to set up new arrangements with head teachers to temporary change their teachers’ schedule, in order to set up a new system focused on shift work. SCEM was encharged with the task of coordinating and supporting the single-seat desks procurement policy.^6^ Despite all the efforts, most processes proceeded slowly and with low-performance indicators: the adaptation of school halls and gyms and the arrangements to borrow extra rooms in other buildings were marginal, the recruitment of new personnel was limited, and the schedule and work of teachers were not changed, thus SCEM had to delay the deadlines set out in the contracts with the production companies. In the end, many students still had to attend lessons at a distance for several months before the necessary two million single-seat desks were distributed to all the eligible schools, and then a part of the supplied desks was of a different size to what had been ordered, and some of these desks even remained in the warehouses.

This policy was not characterized by a complete absence of agility, creativity, and collaboration. However, many setbacks, rigidities, and a lack of alignment with shared goals emerged and contributed to reducing the speed and to worsening the outcomes, despite the huge efforts made by the people involved in the response processes.

When the SCEM staff were tasked with the responsibility of procuring the desks for the reopening of the schools, they worked together with the Direction of the Ministry of Education, which was responsible for contracts and public procurement, and with the Direction responsible for the construction of schools. The SCEM staff discovered that the school system was highly fragmented in the field of the procurement of goods: the desks used in schools had different sizes, high schools purchased desks and other material directly on the market with their own budgets, primary schools received this material from the municipalities, while secondary schools received it from the provinces. Moreover, most production companies were small or medium sized and were only able to satisfy the regional demands. Following the bottom-up governance approach, SCEM allowed the schools, municipalities, and provinces to buy their desks and other necessary teaching materials directly from the suppliers, and only intervened subsidiarily in the case of a lack of supply from the regional markets. However, when many schools and local governments asked for help at the national level, because of the impossibility of obtaining desks directly from firms within a short time, the intervention by SCEM was not resolutive. Analogously, SCEM intervened by distributing personal protection equipment to schools, but again several problems arose, creating stalemates and reducing the speed of the processes. Although some creative ideas emerged, like the one of procuring swivel seats for high schools and that of adapting the already existing stock by sawing double-seat desks into two, they were boycotted right from the beginning by several parties, and the policy therefore proceeded along already beaten paths:

- The desk procurement tenders were formulated by applying the same model used for the procurement of personal protection equipment instead of trying to formulate tenders tailored to this specific challenge;
- The head teachers and their trade union refused to act creatively within the legal framework to adapt school schedules, and also raised supposed insurmountable obstacles;
- The Ministry of education managers were not able to budge from their consolidated procedures.

Although some collaborative dynamics emerged (the grouping of firms to address the challenges of huge selling orders), many other situations were characterized by a lack of collaboration and coordination:

- No preliminary informal meetings or calls were organized with trade associations or the teachers’ trade unions to prepare the production firms and school personnel for the huge challenge of restarting the physical attendance of the students, and the meetings were only arranged after the stalemates, as a reparative strategy;
- In reaction to the SCEM tender, most firms and the main trade association of this productive sector first reacted by boycotting the call and alerting the media about the issue, and then participated in the call but dictated their times to the public administration, thus leading the process to a state of stalemate and substantially forcing SCEM to accept deferred deliveries that lasted until the end of December 2020;
- The schools proved to be inefficient in providing data on the sizes, needs, and requirements of the desks, while the SCEM and the Ministry of Education personnel were reciprocally reluctant to share their own information they had obtained from the territories (about the supply chain, the distribution system, and the personnel recruitment process).

The operative processes proved to be far from agile, probably in part because of this low level of creativity and the collaboration problems. Even though some signs of flexible and prompt strategies to the changing context emerged (the rapid alignment of several firms to the production goal after an increase in prices, and rapid changes in the logistic strategies of some firms), on the whole, the processes were dominated by rigidity and scarce reactivity:

- When SCEM managed to stipulate contracts with several consortiums of firms, the desk production supply chain (mostly involving iron and wood) was not able to boost the production lines;
- Several head teachers sent some face protection mask stocks back to SCEM stating they did not have the space to store them;
- When the head teachers and their trade unions were asked to change schedules and to try to plan work shifts, they raised a number of procedural questions and constraints without offering any information about the possibility of room for maneuver.

When the emotional mechanisms behind these low-robustness processes were examined, it emerged that the fast appraisals of the seriousness of the epidemic and of the necessity of returning to direct school interactions were clear and shared widely among the policy actors. They perceived the situation as a looming disaster, and as a particularly complex and unpredictable situation for which the political and administrative systems and the market were not prepared. Such terms as ‘complex,’ ‘extremely serious,’ and ‘messy’ were used to describe the situation, yet everyone shared the awareness that actions in the education system would have to be taken ‘rapidly,’ ‘immediately.’ Concern and worry were the initial emotions that were framed with such terms as ‘worried,’ ‘confused,’ ‘staggered,’ ‘unprepared,’ ‘lacking clear points of reference and guidelines,’ ‘impotent,’ ‘overcome by the events,’ and ‘fear like that of being in the middle of a storm.’

In reaction to these fast appraisals, the pervasiveness of fear and anxiety was both palpable in the SCEM meetings and explicitly reported during the interviews (one person even used the term ‘panic,’ while only one person explicitly mentioned positive emotions). Fear and anxiety were in particular expressed concerning the risk of doing something that could have been considered illegal or not part of one’s duties or not in line with the contracts, and therefore punishable or liable to blame after the emergency chaos. The fear of ‘making mistakes’ and of ‘tripping up’ was pervasive. As two managers of the education system synthetized:

I think that the fear of taking responsibility for risky actions is blocking or at least slowing down our work: both we and the private organizations are playing a defensive game, because the weight of responsibility is prevailing over our common goals, which, however, are perceived clearly and intimately by all of us, but in the end the potential negative consequences for the individual seem to be prevailing over the potential positive consequences for the community. I don’t know why, but this is actually happening. (I.40)

There was a permanent conference table with the national government and also regional conference tables that periodically gathered together both public managers and private stakeholders of the education system, but the people at these meetings worked badly, and we were in an almost permanent state of panic. (I.92)

The cognitive perceptions of the people involved in this response policy had a rather pessimistic connotation, both during the job shadowing periods and afterward, when these people were asked to remember past events and actions. The development of the situation was framed as unpredictable, changeable, and so uncertain that even the most rigorous scientific estimates and predictions were considered only weakly credible in the eyes of the operators. Most operative decisions, even the ones that could be considered easy in normal times, were perceived to be very risky, and most choices were described as dilemmatic, with options that ran a high risk of failure or negative consequences. Most issues were perceived as foreshadowing tangible risks, and people recognized they had applied more intuition than reflexive thinking to face such issues as choosing between producing new desks and/or cutting the already existing ones, choosing between opening the bids to foreign firms or limiting them to the domestic firms, and the management of the distribution processes to schools. Many people also perceived a low probability of producing something really effective and of reducing the seriousness of the situation because the capacity of individuals was considered ‘inadequate,’ ‘insufficient,’ and ‘too weak to achieve the expected results.’ In this general pessimistic mood, it was comprehensible that most people tended to lay the blame on others for any stalemates, conflicts, polarizations, slowdowns, deviations, and/or weak coordination that arose: SCEM members were biased against school desk companies, the personnel of the Directions of the Ministry of Education were critical about the head teachers, school desk companies were critical about the Public Education Department’s personnel and the special commissioner, and head teachers were suspicious about the national managers’ willingness to take on responsibilities. The reciprocal accusations concerned ‘operative weaknesses,’ ‘the unawareness of the dynamics and constraints of other worlds,’ ‘the existence of hidden objectives,’ and ‘the incapacity to grasp complexity.’ Mention is here made to just two of the reciprocal accusations:

The Ministry of Education is completely lacking in expertise and skills! (I.18)

I remember that the SCEM staff didn’t know anything about the world of school material production and wood production, but they wanted to go on with their rigid convictions. And, indeed, they crashed into a wall (I.41)

The inclination toward the others also proved to be negative and counter-productive: on several occasions, certain people reciprocally deplored the ‘indifference to my arguments,’ the ‘barrage of criticism,’ ‘the incapacity to listen,’ ‘a wall-to-wall situation,’ but, at the same time, most people defended the fact that they ‘were not interested in the preoccupations of others’ because they considered them unfounded and not worthy of attention. The following quotations are representative of the many others that were expressed:

We experienced several conflicts about micro power positions that obscured the relevant objectives and priorities concerning the supply of materials. (I.26)

I wanted them to understand that if I said that something was red, then it was red and there was no way I was going to change my opinion. (I.82)

However, a positive pronounced tendency to privilege straightforward human interactions over formalism also emerged from the reconstruction of the events. This predisposition was framed with the terms ‘frankness’ and ‘informality,’ and with such expressions as ‘more blurred role distinctions,’ ‘more authenticity,’ ‘flattening the hierarchical relations,’ and ‘interacting without beating about the bush.’

On the whole, all the components of the negative emotional mechanism triggered by fear and anxiety were pervasive, except for the presence of a positive predisposition toward informal and more direct interactions. Finally, most people declared they felt a strong sense of impotence and frustration, ‘that left a nasty taste in the mouth,’ as one interviewee mentioned, because of the slowdowns, stalemates, and lack of collaboration, thus confirming the tendency to trigger a vicious circle.

### The procurement of life-saving goods: high robustness and positive emotional mechanisms

The need for personal protection equipment and ventilators far exceeded the capacity of the international market, and a real global commercial war soon emerged to secure stocks of these devices. The competition with other countries was so fierce that several mask orders were blocked at the Italian borders in order to guarantee the supply of the necessary stocks. Despite this extremely serious situation, the Italian response system managed to procure and distribute thousands of ventilators to hospitals, tens of thousands of molecular swabs, and billions of personal protection equipment to the regions every day, and all this in just a few weeks after the declaration of the State of Emergency.

The procurement processes were characterized by a certain agility when faced with the turbulence of the situation.

In the first weeks, the procurement system was based on massive imports of life-saving goods from Asian countries, but it soon became glaringly clear that the international competition was generating several bottlenecks and the Italian surge capacity would have been at risk in just a few weeks. In light of this scenario, the SCEM staff immediately began to set up the necessary processes to boost the domestic production of ventilators and to start up a new domestic production of personal protection equipment, in particular of face protection masks. This meant the huge mobilization of financial and human resources in an extremely short time to start up the R&D processes, to convert firms to new production lines, and to strengthen the whole supply chain. Many obstacles and difficulties arose during these processes. To mention just a few: many firms were really small and were simply unable to produce large quantities of goods; when the market stock of ventilators and personal protection equipment was about to finish, prices soared; the need for ventilators and oxygen in hospitals fluctuated from one day to another because of the unpredictable emergence of hotspots; the logistics and transport system was underequipped, etc. However, the processes proceeded quite rapidly and flexibly to face sudden changes and to adapt to new scenarios:

- The end product companies intervened directly to informally explain and coordinate the changes their supply-chain companies were requested to make to face the fluctuation of the demand and the changing legislation on the standards and features of the products;
- The SCEM staff also decided to start up the production of masks in some prisons, in order to provide masks for convicts and prison personnel, because the situation was serious not only in terms of health risks, but also in terms of physical and psychological safety; the permits, the adaptation of the infrastructures and the professional training of prisoners were all new for the people involved in the management of these processes, but 8 production machines were installed in just a few weeks, while gyms and other spaces were adapted for the new production, without infringing any rules or standards, and specialized training and work were given to almost 300 prisoners in 3 different prisons (for this experience, the Italian case has been considered a best practice by the United Nations Office on Drugs and Crime—UNODC);
- The only ventilator production company was rapidly supported by 25 Army engineers and technicians from a large automotive company, under the coordination of the SCEM staff, to obtain a leap in scale of production and assemblage capacity.

On the whole, all the industrial processes were concluded successfully in about one-third of the time that would usually be needed, with top efficiency indicators: the ventilator company increased its staff by 80% and its production rate quadruplicated in a few weeks. It was therefore able to satisfy the hospital needs, and some of the new face protection mask production machines were ranked among the most efficient and cutting-edge machines on the world market, while almost 100% of the personal protection equipment necessary to protect the Italian population was produced domestically in just a few months and partially distributed for free (to healthcare workers and to students and school personnel).

The supply of molecular swabs also experienced sudden setbacks, and the government promptly intervened at a national level by offering a 50% increase in the surge capacity through the centralized purchase of swabs kits. However, when the SCEM staff began to analyze the situation and to ask regions to specify their requirements, they discovered that the regions had contracts with companies that produced different swab machines, which had been designed to work with different kinds of reagents. At the same time, laboratories within the same region often followed different processing protocols and had different processing capacities. Moreover, some regions had centralized the purchase of healthcare goods, while yet others had decentralized it to the laboratories. When faced with this unexpectedly fragmented landscape, the SCEM staff realized that the predisposition of several different competitive tenders with different timings would have required a great deal of time. In order to speed up the process, SCEM immediately decided to launch an appeal to the medical device trade association to identify the companies that could guarantee a certain amount of supply and to invite them to apply for the upcoming ‘Request for Proposal’ (*Richiesta di Offerta*). Ninety companies applied within a few days, and the SCEM staff proceeded to select those that best fitted the call requisites, with the support of some virologists who were experts in swabs and reagents, and then published the list of companies and products on a shared platform. The regions chose products and quantities in just a few days, and in the end, although with extreme difficulty and even certain controversies, the swabs surge capacity increased by 50% in a few weeks.

Overall, these procurement processes were characterized by a certain degree of creativity:

- In order to speed up the procurement processes, but without disregarding the code of public contracts, the SCEM staff made some informal agreements with companies, who bought several lots of personal protection devices privately and then donated them to the Italian State, and proceeded with the ‘project financing’ formula with several companies of the supply chain to bring part of the production back to Italy, in this way securing Italy not only from the ongoing emergency but also from future possible turbulences;
- The design stage of several products was often conducted in reverse, starting from the materials that were then at the disposal of the companies instead of following the standard project-material-prototype procedure;
- Hundreds of production development stages were substantially tested parallelly by different teams instead of sequentially as usual;
- The production processes were allowed to be initiated immediately, before the authorization offices issued the formal ratifications;
- In order to allow an agile circulation of ventilators in real time according to the hospitals’ needs, SCEM decided to keep hold of the proprietary rights of the devices and to resort to a special form of public borrowing, called Loan for Use (*Comodato d’Uso*), to allow a rapid circulation of ventilators between hospitals.
  Collaboration between the different levels of governments and between public and private institutions was high in most of the procurement processes:
- In order to avoid negative competition mechanisms and equity problems, the national operative committee and the regional chiefs of the CPD, the Committee for the Coordination of the Regions (*Conferenza delle Regioni*), one spokesperson for the Regional Health Minister in each region and some members of the SCEM staff met every day to discuss decisions on the criteria needed to distribute and re-distribute ventilators and personal protection equipment throughout the regions;
- The SCEM staff, the Air force, the National Mail Service, several private transport companies and logistics operators, the regional centers of the CPD, the regional health departments, several hospitals, and many pharmacies collaborated by coordinating the overall logistics and transport system;
- The start-up of the production plants in the prisons proceeded as the result of a close collaboration between public and private organizations that contributed with different expertise: a strategic consultancy company was put in charge of the management of the whole project, while an engineering company worked on the procedures necessary to make some production machines, which had been imported from China, comply with the European and Italian regulations, a temporary worker agency selected the workers who were to train the prisoners on the use of the machines and on safety rules, the Higher Institute of Health certified the masks produced by the prisoners, the administrative permits and the salaries of the prisoners were the duty of the Department of Prison Administration, while SCEM undertook all the necessary expenses and supervised the processes.

The people involved in these processes all shared exactly the same aforementioned serious appraisals of the situation, which was described as ‘an endless chain of disastrous events,’ ‘a war for the procurement of goods,’ ‘a ceaseless flurry of pressing priorities,’ ‘an enormous compression of years into a few weeks,’ and ‘a fight in the dark.’

Stress and worry were high under these adverse conditions, but some positive and productive emotions outweighed the negative feelings. The SCEM members, public officers of national and regional public institutions and agencies, managers and personnel of private firms, NGOs and trade associations were well aware of the fact that the situation was terrible, but they also shared feelings that were framed as ‘pride,’ ‘sense of responsibility,’ ‘sense of importance,’ ‘sense of power’ and, in a few cases, even a ‘sense of gratitude.’ These emotions were reported through various narratives of events, both during the job shadowing periods, when people commented on their feelings at the end of the day, and later on, when people were asked to recount what they remembered about the previous months. These quotations are representative of many others, all of which contained analogous terms and descriptions:

It’s unbelievable, but I feel an inner peace, even though I’m working from early morning to midnight every single day. (…) At the end of the day, I feel relaxed, which is incredible, because I know that I have done all I could do for that single goal together with the other people. (I.3)

When I went to work in the early morning, I somehow liked it. Of course, I was not in the same nightmare condition as the front-line doctors, with patients dying in front of them…I’m aware of this. But we had enormous responsibilities too, we had hospital doctors that were imploring us to help them quickly, we were under the severe eye of the Parliament and of public opinion who checked our decisions and actions critically…and we also knew that we had to be very careful when making rapid decisions because we would have had to give explanations after the emergency. The stress was so high… but I felt active, not lost, because I felt that we were working as a team… a real team. (I.15)

The stress is enormous, but in some ways our organizational well-being… if I can use this term… is at the top. It sounds paradoxical, in a situation in which we all sleep just a few hours and work so hard without any public recognition or monetary compensation for our additional efforts, but I feel good, proud of all this, in some way extremely gratified at the end of the day. (…) Our work takes on a more vivid meaning than what it normally has, even though I usually do a very interesting and fundamental job (I.17)

I felt a sense of shared responsibility, I was not alone in what I was doing; we were on the same boat. And I saw the same feeling in several people with whom I interacted. I think this feeling encouraged people to take one step more instead of one step less.(I.45)

Moreover, surprisingly, some managers and SCEM members who worked on both response policies at the same time reported negative emotions when talking about the single-seat desks procurement processes.^7^

As far as cognitive perceptions are concerned, nobody appeared optimistic about the future, which continued to be framed as unpredictable, changeable, and very uncertain, even after the response processes began to proceed fast and to work effectively. Moreover, many choices were interpreted as dilemmas, as in the case of the single-seat desk procurement processes, and decisions and actions were considered the result of ‘intuition’ or ‘trial and error processes.’ However, almost everybody showed determination and perseverance in dealing with the events, and they explained their capacities as ‘a higher and more effective prioritization than that applied in normal times,’ ‘strength to act in a better and more effective way than usual,’ and ‘capacity to focus on the objective and to put in place prompt strategies.’ The contribution of other people was also considered essential for the success of the emergency operations. Hardly anyone accused the other actors of a lack of commitment, unwillingness to coordinate and cooperate, inexperience, cunning, or bad faith. Confidence and a positive attitude toward the other people involved in response operations were reported by many public and private actors:

It’s as if you reset your priorities to zero; there's a very strong watershed, all your energy is focused only on that problem, and you do not have any other distractions. (I.3)

The urgency and seriousness of the situation gave us the real feeling of the value of time. This made us focus more on the objective. In my opinion, this is what public administration lacks in normal times. (I.15)

Some collaborators of mine are used to protocol documents, but I know they can come up with important skills and, in such a situation, everyone has to maximize their potential, that’s why I leave them the freedom to contribute even to fields outside their usual expertise. And, from the first signals I have seen, my expectations have been rewarded. (I.43)

The collaboration between public and private actors works, because the private sector gives public actors the right push and the public sector helps private actors to act in very complex situations they are not used to. (I. 60)

The attitude toward others proved to be far more positive than for the other policy even among the same people that were involved in both response policies. Moreover, individuals recognized that things began to work well when people ‘began to listen to each other’s needs and perspectives’ and ‘began to understand the worries of the others.’ Parallelly, the tendency to concede and to satisfy others’ requests was also reported by many protagonists through clear expressions of a good predisposition toward compromise or agreement. Just to mention a few of these expressions: ‘when we asked for certain data and information, they answered promptly’(I.28); ‘I saw several people who were well inclined to meet with the others, and our meetings very often ended up with an agreement, which would be incredible in normal times’ (I.37).

Finally, the same disposition to interact more frankly and informally was also widely recognized by the people involved in these processes. This predisposition was framed with the terms ‘stronger concreteness’ and ‘informality,’ and with such expressions as ‘absence of frills,’ ‘suspension of standardized procedures,’ ‘not getting lost in bureaucratic quibbling and formal divisions of competences,’ and ‘weakening individual psychological defenses.’ Some of the people who were re-interviewed some time after the most hectic months were even somewhat nostalgic of this interpersonal predisposition, which did not mean bypassing hierarchy, disregarding rules, or getting ahead of official roles, but basically meant avoiding the rigid observance of convention and/or etiquette.

On the whole, although the perceptions about future developments of the situation were pessimistic and the perception of risk was high, the other components of the positive emotional mechanism triggered by pride were pervasive. Moreover, most people showed and explicitly declared that they felt satisfied, and that they had obtained energy from the collaborative and coordinated interactions and from the relatively fast and flexible solutions that were put in place, thus again confirming the self-reinforcing dynamics of the emotional mechanisms prefigured by the AIM.

## Conclusion

Emergency policies are among the most difficult policies that policy makers have to deal with: a particular combination of extremely challenging factors makes these policies weakly effective and poorly understood (Schneider, 2011). It has been highlighted in scientific literature how some structural factors and organizational strategies (detailed emergency plans, bottom-up multi-level governance systems, and central small multi-expertise coordination teams) can increase the robustness of emergency response policies. However, several studies have also demonstrated that structure and organization cannot fully account for different levels of robustness in response policies, and that cognitive and relational dynamics also play important roles.

In line with psychological literature, which has demonstrated that each decisional process ‘is shaped by momentary emotions in systematic and profound ways’ (Keltner & Lerner, 2010, 330), and in particular when individuals face complex and uncertain situations, this empirical research has attempted to show that also emotions matter and can contribute to explain different levels of robustness in emergency policies.

The affect infusion model has provided the analytical framework that has been used to identify what empirical evidence it was necessary to detect in order to confirm this thesis, while the most similar system design has been applied to compare two response policies to the COVID-19 emergency in Italy. These two policies were similar in terms of timing, complexity, heterogeneity of the decisional arenas, implementation structures, mix of expertise, and in terms of the aforementioned structural and organizational factors, but different in terms of robustness of the decisional and operative processes. In light of the AIM framework, I hypothesized, on one hand, that fear and anxiety tended to be pervasive among the policy makers involved in the emergency response policy that proved to be less robust, and that specific counter-productive cognitive processes and interpersonal predispositions (pessimism about the situation and about individual capacities, systematic perception of having to make dilemmatic choices, tendency to blame other people for negative events, insensitivity to the needs and viewpoints of others and resistance to granting concessions) were dominant in the perceptions and narratives of these policy makers. On the other hand, I hypothesized that pride tended to be pervasive among the policy makers involved in the emergency response policy that registered a higher level of robustness, and that positive cognitive processes and interpersonal predispositions (optimism about the future and about individual capacities, perseverance, tendency to consider other people’s actions as positive contributions to the response processes, sensitivity to the needs and viewpoints of others, and inclination to grant concessions in reaction to other people’s requests) were dominant in the perceptions and narratives of these policy makers.

The research findings have confirmed the two hypotheses. Although starting from challenging and dramatic situations, with very little time, a great deal of uncertainty and high levels of concern and stress, similar structural and organizational conditions, similar appraisals of the seriousness of the situation, and common perceptions of the high risks inherent to the decisions and actions that had to be taken at all costs, the people involved in the two policies experienced somewhat opposite emotional mechanisms. The people involved in the procurement of single-seat desks for the school reopening experienced almost all the negative cognitive and relational dynamics that are typical of the emotional situations characterized by fear and anxiety, while the people who contributed to managing the procurement of personal protection equipment experienced almost all the positive cognitive and relational dynamics that are typical of emotional situations characterized by pride.

In light of these findings, it is possible to state that emotional mechanisms are not able to determine the robustness of policy-making processes in emergency situations, and there remains much fundamental work to be done on the quality of emergency plans and on the good functioning of multi-level governance systems and multi-expertise teams. However, this research suggests that the emotional dimension should not be neglected, from both a theoretical and from a practical perspective, and that preparedness policies might increase their effectiveness, if policy makers were also trained to manage emotions constructively and to put in place strategies to defuse negative counter-productive emotional mechanisms.

## Footnotes

1 For an overview of the impact of the pandemic on several countries and of the national responses, see the Special issue ‘Testing the crisis: opportunity management and governance of the COVID-19 pandemic compared,’ edited by Kuhlmann et al., International Review of Administrative Sciences, 2021, 87(3).

2 The job shadowing periods allowed me to follow the emergency response processes directly and to observe what nobody was allowed to see from the outside.

3 To maintain confidentiality, and according to the express will of the respondents, the identities of the interviewees have been omitted and are referred to in the text with only letters and progressive numbers. The number exceeds 41 because the interviews on the two selected response policies were conducted contemporarily with the interviews on other response policies.

4 The so-called carryover (Bodenhausen, 1993; Loewenstein and Lerner, 2003) of incidental emotions consists in transposing, into a situation, an emotion generated by a source (situational or environmental) that has nothing to do with the situation in question, often with the people who experience it having no awareness of the mechanism. ‘For example, incidental anger triggered in one situation automatically elicits a motive to blame individuals in other situations even though the targets of such anger have nothing to do with the source of the anger’ (Lerner et al., 2015, p. 803).

5 https://www.salute.gov.it/imgs/C_17_pubblicazioni_501_allegato.pdf

6 The Minister’s decision was contested in Parliament and became controversial in the media debate, but in the opinion of many experts and practitioners, desks were considered a fundamental tool to allow classroom activities in the Italian primary and secondary schools to resume, because the education system is still largely centered on traditional face-to-face and non-technological teaching methods.

7 It was precisely this divergence in the reconstructions of the events and feelings during the first job shadowing periods at SCEM that convinced me to write an article on the role of emotions in emergency management policies.
